# P-I metalloproteinases and L-amino acid oxidases from *Bothrops* species inhibit angiogenesis

**DOI:** 10.1590/1678-9199-JVATITD-2020-0180

**Published:** 2021-08-18

**Authors:** Shreesha K. Bhat, Manjunath B. Joshi, Sampara Vasishta, Rajesh N. Jagadale, Setlur G. Biligiri, Monika A. Coronado, Raghuvir K. Arni, Kapaettu Satyamoorthy

**Affiliations:** 1 Manipal School of Life Sciences, Manipal Academy of Higher Education, Manipal, India.; 2 Juggat Pharma, Jagadale Industries, Bangalore, India.; 3 Multiuser Center for Biomolecular Innovation, Department of Physics, São Paulo State University (UNESP), São José do Rio Preto, SP, Brazil.

**Keywords:** P-I metalloproteinases, LAAO, Bothrops, Anti-angiogenic, Snake venom

## Abstract

**Background:**

Snake venoms are composed of pharmacologically active proteins that are evolutionarily diverse, stable and specific to targets. Hence, venoms have been explored as a source of bioactive molecules in treating numerous diseases. Recent evidences suggest that snake venom proteins may affect the formation of new blood vessels. Excessive angiogenesis has been implicated in several pathologies including tumours, diabetic retinopathy, arthritis, *inter alia*. In the present study, we have examined the effects of P-I metalloproteinases isolated from *Bothrops moojeni* (BmMP-1) and *Bothrops atrox* (BaMP-1) and L-amino acid oxidases (LAAO) isolated from *B. moojeni* (BmLAAO) and *B. atrox* (BaLAAO) on biochemical and functional aspects of angiogenesis.

**Methods:**

P-I metalloproteinases and LAAO were purified from venom by molecular size exclusion and ion-exchange chromatography and subsequently confirmed using mass spectrometry. The P-I metalloproteinases were characterized by azocaseinolytic, fibrinogenolytic and gelatinase activity and LAAO activity was assessed by enzyme activity on L-amino acids. Influence of these proteins on apoptosis and cell cycle in endothelial cells was analysed by flow cytometry. The angiogenic activity was determined by *in vitro* 3D spheroid assay, Matrigel tube forming assay, and *in vivo* agarose plug transformation in mice.

**Results:**

P-I metalloproteinases exhibited azocaseinolytic activity, cleaved α and partially β chain of fibrinogen, and displayed catalytic activity on gelatin. LAAO showed differential activity on L-amino acids. Flow cytometry analysis indicated that both P-I metalloproteinases and LAAO arrested the cells in G0/G1 phase and further induced both necrosis and apoptosis in endothelial cells. *In vitro,* P-I metalloproteinases and LAAO exhibited significant anti-angiogenic properties in 3D spheroid and Matrigel models by reducing sprout outgrowth and tube formation. Using agarose plug transplants in mice harbouring P-I metalloproteinases and LAAO we demonstrated a marked disruption of vasculature at the periphery.

**Conclusion:**

Our research suggests that P-I metalloproteinases and LAAO exhibit anti-angiogenic properties *in vitro* and *in vivo*.

## Background

Snake venoms are a complex mixture of physiologically active proteins that makes them attractive sources of therapeutically important molecules [1]. Snake venom proteins are multifunctional, evolutionarily diverse and possess a high degree of effectiveness and stability. These properties are inviting to explore and develop novel compounds for therapeutic purposes [2]. Snakes of *Bothrops* genus, belonging to Viperidae family*,* are abundantly spread over Brazil and neighbouring countries [3]. More than 90% of proteins from *Bothrops* venom have been reported to interfere with several physiological processes [4]. Envenoming by *Bothrops* spp. is characterized by local tissue damage that includes hemorrhage, pain, edema and necrosis, and may also cause systemic disruptions such as coagulopathies and renal failure [5,6]. Major proteins of venom from Viperidae family include serine proteinases, metalloproteinases, phospholipases and L-amino acid oxidases (LAAO).

Snake venom metalloproteinases (SVMP) belong to a subfamily of enzymes called reprolysin with an ability to cleave selective key peptide bonds. Proteomic analysis showed the presence of multifunctional metalloproteinases in the venom of Crotalid and Viperid snakes [7]. SVMPs are large multidomain proteins synthesized in the venom gland with pro-enzyme domain and a conserved zinc-protease domain [8]. The secreted enzymes are categorized into P-I mature (metalloproteinase domain), P-II (metalloproteinase domain, disintegrin-like) and P-III (metalloproteinase domain, disintegrin-like, cysteine-rich) [9,10,11]. Several aspects of snake venom metalloproteinases (SVMP) with potential pathological effects have been studied. These includes vascular damage leading to hemorrhage [12], necrosis and poor regeneration of skeletal muscle [13], dermonecrosis and blistering [14] and the inflammatory molecules responsible for edema, pain and chemotaxis [15,16]. Jararhagin, a P-III metalloproteinase isolated from *B. jararaca*, has been widely studied for fibrinolytic action, hemorrhagic activity and degradation of ECM proteins [17]. Similarly, P-I metalloproteinases were purified and characterized with high catalytic activity on fibrin [18], kininogen [19] and degradation of extracellular matrix [20]. P-I metalloproteinases showed hemorrhagic activity [14], weak [21] or non-hemorrhagic activity [22], platelet aggregation [23], disintegration activity and apoptosis on endothelial cells by the process anoikis [24]. During envenomation, endothelial damage caused by metalloproteinases leads to poor tissue regeneration. Earlier studies have demonstrated that SVMP alter structure and function of endothelial cells [11].

L-amino acid oxidase (LAAO) is a flavoenzyme that catalyzes the oxidative deamination of L-amino acid substrate into an α-keto acid along with the formation of hydrogen peroxide (H_2_O_2_) and ammonia (NH_3_). The snake venom LAAO depends on flavin adenine dinucleotide (FAD) molecule and is responsible for the toxicity as a consequence of H_2_O_2_ produced during the transiently reduced flavin cofactor reoxidation by molecular oxygen [25,26]. LAAO, a major toxin present in the viper venom, have been demonstrated to possess cytotoxic activity. Using a battery of cell and biochemical assays *in vitro*, studies have shown anti-tumour properties of snake venom-LAAO (SV-LAAO) in various cancer cell lines such as MCF-7 and SKBR-3 (breast adenocarcinoma cells) [27,28] and melanoma [29] S-180 (murine sarcoma 180) [27]. LAAO are also capable of inducing apoptosis on endothelial cells [30], leukemic cells [31], keratinocytes [32] and other several cells types by inducing oxidative stress, which is critical in supressing the tumour growth. Over the years, studies have indicated that reactive oxygen species (ROS) damage the cell membrane lipids and may cause potential cytotoxicity [33,34]. As cellular membrane of tumour cells are composed of higher levels of lipids compared to the normal cells, H_2_O_2_ produced by SV-LAAOs may cause a direct and specific effect on tumour cells [35].

Angiogenesis is one of the key processes involved in the remodelling of the tissue microenvironment. Tissue remodelling is a complex process that involves the development of new capillaries from pre-existing blood vessels. Angiogenesis engage in various normal physiological processes, such as wound healing, female menstrual cycle and bone remodelling. In contrast, several pathological conditions are also accompanied with undesirable neovascularization, including tumour growth, metastasis, diabetic retinopathy and inflammatory diseases [36]. Angiogenesis is a pre-requisite in solid tumours to enable their growth and metastasis. Therefore, impeding the growth of blood vessels in the tumour microenvironment comprises an attractive therapeutic strategy. Accordingly, identification of molecules showing anti-angiogenic properties have gained clinical importance [37]. Several biomolecules exhibiting anti-angiogenic activity are currently being studied, naturally including few components of snake venom [38].

Hence, in the present study we have (i) isolated the P-I metalloproteinases (BmMP-1 from *B. moojeni* and BaMP-1 from *B. atrox*) and LAAO (BmLAAO from *B. moojeni* and BaLAAO from *B. atrox*) proteins from venom, (ii) characterized these proteins by batteries of biochemical activities and (iii) examined their ability to modulate angiogenesis *in vitro* and *in vivo*.

## Methods

### Chemicals

Azocaesin, methylcellulose and fibrinogen were obtained from Sigma-Aldrich (St Louis, USA). Collagen R solution, Matrigel and basic fibroblast growth factor (bFGF) were obtained from SERVA electrophoresis (Heidelberg, Germany), BD biosciences (San Jose, USA) and R & D systems (Minneapolis, USA) respectively. The crude venom samples of *B. moojeni* and *B. atrox* were purchased from San Maru Serpentarium, Brazil, and their use was approved by the Brazilian Institute of the Environment andef Renewable Natural Resources (Ibama). Purification columns Superdex 200 column and Mono Q 5/50 were purchased from GE Healthcare (Little Chalfont, UK).

### Molecular size exclusion chromatography

*Bothrops* venom (~25 mg) was separately dissolved in 0.6 mL of a 0.02 M sodium acetate buffer pH 7.3 containing 0.1 M NaCl and centrifuged at 10,000× g for 5 minutes. The supernatant was applied into a Superdex 200 column, previously equilibrated with the same buffer. The protein fractions were collected every minute at the flow rate of 0.4 mL/min, which was monitored at 280 nm.

### Ion-exchange chromatography

The fractions containing P-I metalloproteinase and LAAO from molecular size exclusion chromatography were pooled separately and applied onto a Mono Q 5/50 GL column, previously equilibrated with 0.02 M Tris-HCl pH 8.2 and 0.02 M HEPES pH 7.5 buffers, respectively. The bound protein fractions were eluted with a non-linear gradient with 0.02 M Tris-HCl pH 8.2 containing 1 M NaCl with the flow rate of 0.5 mL/minute, monitored at 280 nm. All the peak fractions were concentrated, and the purity was evaluated by 10% sodium dodecyl sulphate polyacrylamide gel electrophoresis (SDS-PAGE).

### Peptide mass fingerprinting of P-I metalloproteinases and LAAO

Peptide fingerprints of P-I metalloproteinases and LAAO were determined using liquid chromatography/mass spectrometry as described in our earlier [39]. The purified protein was run on SDS PAGE and was stained with Coomassie brilliant blue R-250. The protein bands were sliced, treated with 5 mM DTT and the gel pieces were immersed in 55 mM iodoacetamide and incubated in the dark for 30 minutes. This was followed by washes with ammonium bicarbonate (NH_4_HCO_3_) and acetonitrile. The gel pieces were vacuum dried and incubated with 5 mg/mL trypsin in NH_4_HCO_3_ and incubated at 37º C overnight. Subsequently, the peptide extraction was performed stepwise by treating the gel pieces with (i) 30% acetonitrile in 1% trifluoro acetic acid (TFA), (ii) 1% TFA and (iii) 70% acetonitrile with 1% TFA. In each step, 30 minutes of vortex and 3 minutes of sonication were performed before collecting the supernatant. Supernatant collected from each step was combined, dried in vacuum and reconstituted with acetonitrile. Tryptic digested sample (8 µL) was injected into HPLC coupled with Agilent 6520 accurate-mass Q-TOF LC/MS using a reverse phase eclipse plus C18 column, 5 μm, 4.6 mm X 150 mm (Agilent Technologies, Santa Clara, US) and peptides were eluted in gradient form with 30-70% acetonitrile gradient. MS/MS analysis was performed, peaks obtained were processed and converted to Mascot generating file (.MGF) using Qualitative Mass Hunter (Agilent Technologies). The .MGF files were processed in Mascot database version 2.3 (Matrix Science Limited, London, UK). Proteins were identified using the following parameters: enzyme as trypsin, carbamidomethylation as fixed modification, methionine oxidation as variable modification and with peptide charges more than 3+. Peptide blast was performed using Swiss Prot database.

### Azocaseinolytic activity of metalloproteinases

For characterization of P-I metalloproteinases, either BmMP-1 or BaMP-1 (2 µg) was incubated with azocasein (1.5 mg/mL) in 0.5% Sodium carbonate containing 5 mM CaCl_2_ and incubated at 37ºC for 60 minutes [40]. Further, 100 μL of 20% (v/v) trichloroacetic acid (TCA) was added and incubated for 30 minutes on ice, followed by centrifugation for 15 minutes. For 1 mL of supernatant, 500 µL of 1 M NaOH was added and absorbance of the supernatant was determined at 405 nm using Varioskan™ LUX multimode microplate reader (Thermo Fisher Scientific, USA). Further, the effect of inhibitors (EDTA, EGTA, PMSF, 1,10 Phenanthroline, CaCl_2_, ZnCl_2_, MgCl_2_ and HgCl_2_), (10 mM, each) was tested on P-I metalloproteinases by incubating for 30 minutes at 37ºC.

### Fibrinogenolytic activity

The fibrinogenolytic activity of BmMP-1 or BaMP-1 (2 µg/mL) was determined by incubating with 10 µg of fibrinogen in 0.1 M Tris-HCl buffer pH 7.4 (30 µL reaction mixture) for 2 h at 37ºC [41]. Laemmli buffer was added to stop the reaction and analysed on 10% SDS PAGE for the degradation of sub-units of fibrinogen.

### Gelatinase activity

To assess gelatinase activity, either BmMP-1 or BaMP-1 (1 µg/lane) were separated on 10% SDS PAGE containing 0.3% gelatin as a substrate [41]. The gel was incubated for 90 minutes with renaturation buffer (0.5% Triton X-100) and developing buffer (10 mM Tris pH 7.8, 5mM CaCl_2_, 100mM NaCl) for 16-20 h. Further, the gel was stained with Coomassie brilliant blue G-250 and the activity was observed for a clear zone in the gel.

### Enzyme activity of LAAO

The LAAO activity was measured by the initial rate of H_2_O_2_ production with peroxidase/dye assay [42]. The assay mixture contained either BmLAAO or BaLAAO (2 µg), 0.01 M L-amino acid, 0.2 M Tris-HCl buffer pH 8.0, 0.2 mg/mL o-dianisidine hydrochloride, 1 U/mL horseradish peroxidase. Further, the reaction mixture was incubated at 30°C for 1 h and the dye formation was measured spectrophotometrically at 410 nm in a Varioskan™ LUX multimode microplate reader (Thermo Fisher Scientific, USA). Further, the effect of divalent metal ions (CaCl_2_, ZnCl_2_, MgCl_2_, HgCl_2_ - 10 mM each) and amino acid derivative N-acetyl cysteine (5 mM), was tested on LAAO activity using L-Leu, L-Met, L-His, L-Tyr and L-Trp as substrates by incubating for 30 minutes at 37ºC.

### Cell culture

Endothelial cells (HUVECs) were cultured in Endothelial Cell Growth Medium (ECGM) along with endothelial supplements containing 5% Fetal bovine serum (FBS - Promo cell GMBH, Germany) on gelatin coated plates maintained at 37°C with 5% CO_2_. Fibroblast cells cultured in DMEM supplemented with 10% FBS (HiMedia, India) at 37°C with 5% CO_2_ as described previously [39]_._ HepG2 cells (ATCC) were cultured in DMEM 10% FBS.

### MTT assay

MTT based cytotoxicity assay was performed on HUVECs, fibroblasts and HepG2. The cells were seeded at the density of 10,000 cells/well in a 96 well plate. The endothelial cells were treated with different concentration BmMP-1 or BaMP-1 (0.5 μg/mL, 1 μg/mL, 2 μg/mL, 3 μg/mL) or BmLAAO or BaLAAO (1 ng/mL, 10 ng/mL, 100 ng/mL, 200 ng/mL) or mitomycin (50 μg/mL) maintained at 37°C with 5% CO_2_ for 24 h. For Fibroblast and HepG2 treated with BmMP-1 or BaMP-1 (2 μg/mL) and BmLAAO or BaLAAO (100 ng/mL) maintained at 37°C with 5% CO_2_ for 24 h. After the incubation, 20 μL of 5 mg/mL MTT was added and incubated for 4 h. DMSO (100 μl) was added to dissolve Formazan crystals and absorbance 570 nm was noted using Varioskan^TM^ flash multimode reader (Thermo Scientific, USA).

### Three-dimensional collagen spheroid assay

Endothelial cells were resuspended in basal ECGM containing 20% of methylcellulose. The cell solution was dispensed on non-cell culture treated surface of petri dishes to obtain 5000 cells/drop and incubated at 37ºC with 5% CO_2_ for 24 h. The spheroids formed in each drop were implanted within the collagen matrix with different concentrations of BmMP-1 or BaMP-1 (0.5 µg /mL, 1 µg/mL and 2 µg/mL)/BmLAAO or BaLAAO (1 ng/mL, 10 ng/mL and 100 ng/mL)/bFGF (10 ng/mL)/PBS and cultured for 24 h. The length of sprouts per spheroid was measured morphologically by using ImageJ software (NIH, USA).

### Tube forming assay

Matrigel (50 µL) was coated on 96-well plate allowed to solidify at 37ºC for 1 h. HUVECs were seeded on Matrigel cushion at the density of 2 x 10^4^ cells/well. Then the cells were treated with 2 µg/mL of BmMP-1 or BaMP-1, 100 ng/mL of BmLAAO or BaLAAO, 10 ng/mL of bFGF and PBS (Control) and incubated at 37°C with 5% CO_2_ for 6 h. Kinetics of tube formation at different time points was observed and counted.

### Cell cycle analysis

The HUVECs were seeded onto 6 well plate at the density of 10^5^ cells/mL and incubated to grow to 80% confluency. The HUVECs were washed twice with PBS and serum starved (0.5% FBS) for 4 h. Further, the cells were treated with 2 μg/mL of BmMP-1 or BaMP-1 and 100 ng/mL of BmLAAO or BaLAAO for 12 h. After the incubation, the cells were trypsinized and centrifuged for 3 minutes. Single cell suspension was fixed upon adding 70% ethanol drop by drop and incubated in 4ºC for 1 h. The pellet was washed with PBS and RNAase (0.5 μg/mL) was added to the pellet and further incubated at 37(C for 1 h. Propidium iodide (10 μg/mL) in PBS was added to the pellet and incubated on ice for 20 minutes. This suspension was passed through flow cytometer (Sysmex Partec, Germany) and data were analysed using cyFlow software (Sysmex Partec, Germany).

### Annexin V/propidium iodide (PI) staining

The HUVECs were treated as mentioned in cell cycle analysis. After treatment, the cells were trypsinized and centrifuged for 3 minutes for 800 rpm for 5 minutes. Further, Annexin V analysis was performed using FITC Annexin V Apoptosis Detection Kit (BD Biosciences, 556547). This suspension was passed through flow cytometer (Sysmex Partec, Germany) and data were analysed using cyFlow software (Sysmex Partec, Germany).

### Agarose plug transplantation assay

The animal experiments were conducted upon approval from Institutional Animal Ethics Committee (IAEC), Manipal Academy of Higher Education, Manipal (IAEC/KMC/48/2015). Animal handling in the present study was accordance with the standard guidelines of the committee for the purpose of control and supervision on experiments on animals (CPCSEA), Government of India. The animals were nurtured in sterile polypropylene cages and maintained in control conditions as follows: Temperature 23 ( 2(C, humidity 50 ( 5% and 12 h light/dark cycle, access to sterile water and food ad libitum. The agarose plug transplantation assay was performed as described earlier [24], to demonstrate the effect of P-I metalloproteinases and LAAO proteins on angiogenesis *in vivo*. Swiss albino mice (6-8 weeks, 25-30 g) were randomly used in six groups; control, bFGF, BmMP-1, BaMP-1, BmLAAO and BaLAAO (n = 6 in each group). Before the insertion of agarose plug, mice were anesthetized with Ketamine-anket (65 mg/kg) and Diazepam-Calmpose (8 mg/kg). BmMP-1 or BaMP-1 (5 µg/mL)/BmLAAO or BaLAAO (1 µg/mL)/bFGF (100 ng/mL)/PBS was mixed with agarose gel (2%, 50 μL) and was allowed to solidify. Then the agarose pellet was introduced into subcutaneous space of mice under sterile condition and the animals were in-housed for five days in our institutional animal house in central animal research facility (CARF), following the animal was euthanized with standard procedure. Further, the tissues were excised and photographed with the surrounding skin.

### Statistical analysis

All the experiments were performed in triplicates independently. Unpaired two-tailed Student t-test and ANOVA were performed with or without *post-hoc* tests using GraphPad Prism (Version 8). Continuous data were represented as mean ± standard deviations (SD) and p-value < 0.05 was considered as a significant change.

## Results

### Purification and characterization of P-I metalloproteinases and LAAO

**Figure 1.**
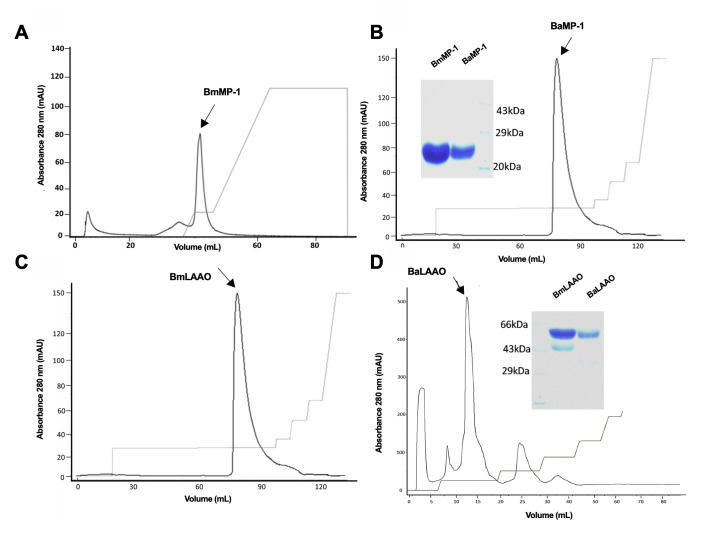
Purification of P-I metalloproteinases and LAAO from *Bothrops* venom. Peaks from size exclusion chromatography related to P-I metalloproteinases and LAAO were applied to the column and elution was carried out with NaCl gradient buffer. Peaks corresponding to respective proteins **(A)** BmMP-1, **(B)** BaMP-1, **(C)** BmLAAO and **(D)** BaLAAO are denoted by arrows. Inset: SDS-PAGE analysis of BmMP-1 and BaMP-1 and BmLAAO and BaLAAO.

P-I metalloproteinases and LAAOs were purified from crude venoms of *B. atrox* and *B. moojeni* by molecular size exclusion and ion-exchange chromatography. Figure 1 shows the separation of P-I metalloproteinases and LAAO by ion-exchange chromatography. P-I metalloproteinases isolated from *B. moojeni* (BmMP-1) and *B. atrox* (BaMP-1) showed molecular weight of ~25 kDa on SDS PAGE (10%) which is in agreement with P-I metalloproteinases isolated from *Bothrops* spp. in earlier studies as summarized in Table 1 (Fig. 1A and 1B). The molecular weight of LAAO isolated from *B. moojeni* (BmLAAO) and *B. atrox* (BaLAAO) was ~58 kDa on SDS PAGE (10%) which is in accordance with the protein isolated from different *Bothrops* spp. (Table 2) (Fig. 1C and 1D).

**Table 1.** Comparison of biochemical properties of P-I metalloproteinases isolated from different snakes.

Protein	Species	MW	Azocasein	Fibrinogenolytic	Gelatinolytic	Reference
BaP1	*B.asper*	25	+	+	-	[49]
Crotalin	*Crotalus atrox*	25	-	+	-	[74]
Graminelysin I	*Trimeresurus gramineus*	25	+	+	-	[68]
Triflamp	*Trimeresurus flavoviridis*	25	+	+	-	[75]
BmooMPa-I	*B. moojeni*	24.5	+	+	-	[47]
Batx-I	*B. atrox*	23.29	+	+	-	[43]
BmooMP-I	*B. moojeni*	~25	-	+	+	[48]
Botroxase	*B. atrox*	22.9	-	+	-	[76]
BpirMp	*B. pirajai*	23.1	-	+	-	[77]
BmMP-1	*B. moojenoi*	25	+	+	+	Present study
BaMP-1	*B. atrox*	25	+	+	+	Present study

+ Determined; - not determined.

**Table 2.** Comparison of functional properties of LAAO isolated from different *Bothrops* spp.

Enzyme activity of LAAO					
Amino acids	Bpir LAAO-1, [58] (MW - 66 kDa)	Bp-LAAO, [78] (MW - 58 kDa)	Lm LAAO, [79] (MW - 60 kDa)	BmLAAO, Present study (MW - 58 kDa)	BaLAAO, Present study (MW - 58 kDa)
L-Ala	🡺	🡻	ND	🡻	🡻
L-Gly	🡻	ND	ND	🡻	🡻
L-Ile	🡹	🡹	🡹	🡻	🡺
L-Leu	🡹	🡹	🡹	🡹	🡹
L-Pro	🡺	🡻	ND	🡻	🡻
L-Val	🡹	🡺	🡺	🡹	🡺
L-Phe	🡹	🡹	🡹	🡹	🡺
L-Trp	🡹	🡹	🡹	🡹	🡺
L-Tyr	🡹	🡺	🡹	🡹	🡺
L-Asp	ND	ND	ND	ND	ND
L-Glu	🡻	🡻	ND	ND	ND
L-Arg	🡺	🡹	🡺	🡺	🡺
L-His	🡹	🡹	🡺	🡹	🡹
L-Lys	🡻	ND	🡻	🡺	🡻
L-Ser	🡻	🡻	🡻	🡻	🡻
L-Thr	🡻	🡻	ND	🡻	🡻
L-Cys	ND	🡻	🡻	🡻	🡻
L-Met	🡹	🡹	🡹	🡹	🡹
L-Asn	ND	ND	ND	🡺	🡻
L-Gln	ND	ND	ND	🡻	🡻

🡹 High activity; 🡺 moderate activity; 🡻 low activity; ND: not determined.

Further, P-I metalloproteinases and LAAO were characterized by mass spectrometry analysis. Positive mode ionization of BmMP-1 detected eight peptides matching to VM3BOP protein of *B. jararaca* with 11% coverage and a score of 422. BaMP-1 detected five peptides matching to that of *B. jararaca* with 30% coverage and a score of 784 as shown in Table 3. For BaLAAO*,* we obtained ten peptides with precise homology to LAAO of *B. jararaca* with coverage of 40% and possessing a highly significant score of 2088. Further, BmLAAO detected nine peptides homologous to *B. moojeni*, with a score of 572 and coverage of 30% as shown in Table 4.

**Table 3.** Peptide mass fingerprinting of BmMP-1 and BaMP-1.

Sample	Mascot score	Protein name	Accession ID	Observed peptide sequence	MW (Da)	Sequence coverage
BmMP-1	422	VM3BP_BOTJA	O93523	ARMYELANIVNEILR SGSQCGMGDCCEQCK SGTECRASMSECDPAEKCTGQSSECPADVFHK GNYYGYCR KIPCAPEDVK DNSPGQNNPCK GNVLPGTKCADGKVCSNGMCVDVATAY	70392	40%
BaMP-1	784	VM3BP_BOTJA	O93523	ITVKPDVDYTLNSFAEWRKTDLLTR SGTECR GNYYGYCR KIPCAPEDVK DNSPGQNNPCK GMVLPGTKCADGK	70392	11%

**Table 4.** Peptide mass fingerprinting of BmLAAO and BaLAAO.

Sample	Mascot score	Protein name	Accession ID	Observed peptide sequence	MW (Da)	Sequence coverage
BaLAAO	2088	OXLA_BOTJR	Q6TGQ9	NPLEECFR NGLSTTSNPK EGWYANLGPMR VGEVNKDPGVLDYPVKPSEVGKSAGQKAVEELRR YDTYSTKEYLLK MDDIFAYEKRFDEIVGGMDKLPTSMYQAIAEKVKLHAR IQQDVKEVTVTYQTSEK IKFEPPLPPKKAHALRSVHYR I FLTCTKKFWE	56595	40%
BmLAAO	576	OXLA_BOTMO	Q6TGQ8	ETDYEEFLEIAK EGWYANLGPMRVGEVNKDPGVLEYPVKPSEVGKSAGQLYEESLQKAVEELR YDTYSTKEYLLK HDDIFAYEK FDEIVGGMDKLPTSMYQAIAEK IQQDVK KAHALR IFLTCTKKFWEDDGIHGGKSTTDLPSR	54858	30%

### Biochemical characterization of P-I metalloproteinases

**Figure 2.**
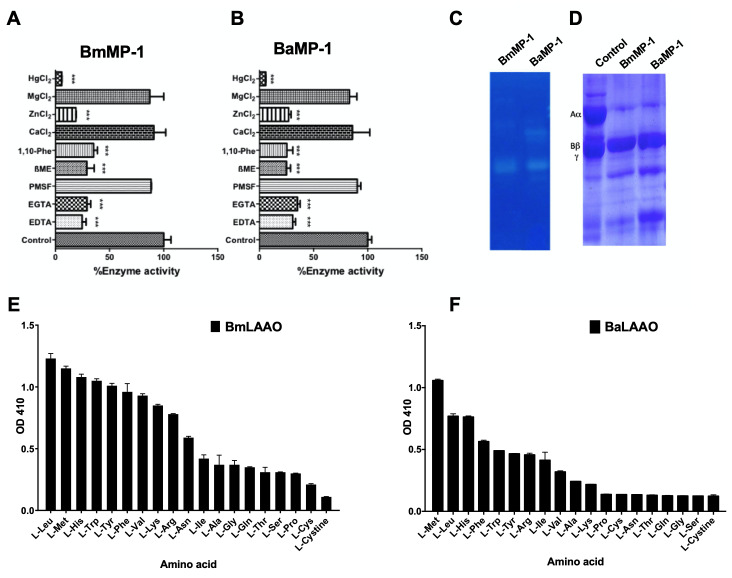
Biochemical characterization of P-I metalloproteinases and LAAO. **(A)** and **(B)** Azocaseinolytic activity of BmMP-1 and BaMP-1. Effect of inhibitors (10 mM), metal chelators (EDTA and EGTA), divalent metals (CaCl_2_, ZnCl_2_, MgCl_2_ and HgCl_2_), protease inhibitors (1,10 phenanthroline and PMSF) and reducing agent (β-mercaptoethanol) on azocaseinolytic activity in the presence of BmMP-1 or BaMP-1. The data are represented as percentage of untreated control and statistical significance is indicated by asterisk ***p < 0.001. **(C)** BmMP-1 or BaMP-1 degraded gelatin on SDS-PAGE indicted by zone of clearance. **(D)** Fibrinogen (10 μg) was treated with BmMP-1 or BaMP-1 and degradation of α chain and partial degradation of β chain were observed on SDS-PAGE. L-amino acids (10 mM) were treated with **(E)** BmLAAO (2 μg) or **(F)** BaLAAO (2 μg) coupled with o-dianisidine HCL/HRP. The activity was measured spectrophotometrically at 410 nm. The data are shown as arbitrary units.

We characterized P-I metalloproteinases isolated from *Bothrops* spp. by caseinolytic assay using azocasein as the substrate. P-I metalloproteinases isolated from *B. moojeni* and *B. atrox* significantly hydrolysed the azocaesin. However, BmMP-1 exhibited the highest activity when compared to BaMP-1. Further, the metalloproteinase activity was inhibited by metal chelators such as EDTA and EGTA and also protease inhibitor 1, 10 phenanthroline. However, PMSF, another class of protease inhibitor did not affect the activity of the protein. Divalent metal ions such as Zn^+2^ and Hg^+2^ were able to inhibit 40-50% of the activity, but Mg^+2^ and Ca^+2^ did not show any effect. β-mercaptoethanol significantly inhibited the activity of protein, suggesting that reduced activity may be due to the changes in the conformation of the protein due to the reduction of cysteine disulfide bonds (Fig. 2A and 2B).

To assess the gelatinolytic activity of P-I metalloproteinases, we performed gelatin zymography. BmMP-1 and BaMP-1 showed a zone of clearance on gelatin gel signifying potential gelatinolytic activity (Fig. 2C). Our analysis revealed that P-I metalloproteinases from *B. atrox* and *B. moojeni* cleaved α chain and partially β chain of fibrinogen (Fig. 2D) (Table 1).

### Biochemical characterization of LAAO

The LAAO activity was determined by quantifying the initial rate of H_2_O_2_ production in the combination of peroxidase/dye assay. The activity of the protein was proportionate to the formation of dye by the catalytic activity of the protein, which was then read at 410 nm. The assay revealed that LAAO isolated from both species had displayed differential enzyme activity. BmLAAO showed high specificity towards L-Leu L-Met L-His L-Trp L-Tyr L-Phe L-Val, moderate to L-Lys L-Arg L-Asn and low specificity to L-Ile L-Ala L-Gly L-Gln L-Thr L-Ser L-Pro L-Cys L-Cystine; BaLAAO exhibited had high specific activity towards L-Met, L-Leu, L-His, moderate activity towards L-Phe, L-Tyr, L-Arg, L-Ile, L-Val and low activity towards L-Ala, L-Lys, L-Pro, L-Cys, L-Asn, L-Thr, LGln, L-Gly L-Ser, L-Cystine (Fig. 2E and 2F) (Table 2). This indicated that the specificity towards amino acid substrates varied from two different species of *Bothrops*. Further, we examined inhibotory effects of CaCl_2_, ZnCl_2_, MgCl_2_, HgCl_2_ and N-acetyl cysteine on LAAO activity for substartes with highest specificites. We observed only ZnCl_2_, HgCl_2_ and N-acetyl cysteine showed significant inhibition for LAAO activity (Additional file 1A and 1B). 

### P-I metalloproteinases and LAAO induce both necrosis and apoptosis in endothelial cells

To understand toxicity of P-I metalloproteinases and LAAO, we performed MTT assays. Upon culturing fibroblasts for 24 h, we observed that P-I metalloproteinases and LAAO induced approximately 20% and 20-30% cell death respectively. These proteins were more toxic to endothelial cells than fibroblasts and HepG2. P-I metalloproteinases induced cell death of nearly 40% whereas LAAO stimulated 50% cell death after 24 h. This suggests that metalloproteinases and LAAO derived from *Bothrops* spp. possess cell specific effects (Additional file 2A, 2B and 2C).

**Figure 3.**
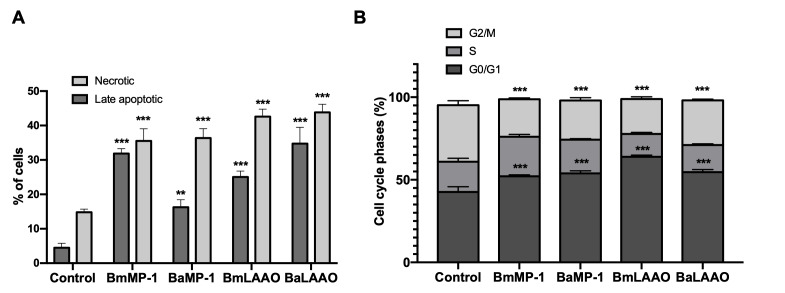
Influence of P-I metalloproteinases and LAAO on apoptosis and cell cycle. The endothelial cells were treated with BmMP-1 or BaMP-1 (2 μg/mL) and BmLAAO or BaLAAO (100 ng/mL) and processed for flow cytometry analysis to determine **(A)** mode of cell death and **(B)** (re)distribution of cells into cell cycle phases. **(A)** Cells were stained with annexin V conjugated with FITC and propidium iodide and analysed in a flow cytometer. Data are represented in percentage for necrotic and late apoptotic cells *versus* control. Statistical significance is represented by asterisk, ***p < 0.001 and **p < 0.01 *versus* control. **(B)** After treating cells as indicated above, they were fixed in ethanol and stained with PI and analysed by flow cytometry. Data are represented in percentage for G0/G1 (arrest/preparation), S (DNA synthesis) G2/M (mitosis preparation) against control. Statistical significance is represented as asterisk ***p < 0.001.

Further, we performed flow cytometry analysis to distinguish apoptosis and necrosis by staining phosphatidylserine with annexin V. HUVECs were treated with P-I metalloproteinase and LAAO for 12 h. The treatment on endothelial cells with both proteins showed a significant decrease in the viable cells and promoted large number of cells at late apoptotic phase of 16% and 31% in presence BmMP-1 and BaMP-1 respectively. Similarly, BmLAAO and BaLAAO induced apoptosis of 34% and 25% respectively. PI stained cells indicated necrosis of significant proportion of cells at 35% (BmMP-1) and 36% (BaMP-1) by P-I metalloproteinases and 42% (BmLAAO) and 43% (BaLAAO) by LAAO. These results indicated that P-I metalloproteinases and LAAO induced both necrosis and late apoptotic mode of cells death (Fig. 3A, Additional file 3B).

### P-I metalloproteinases and LAAO facilitated significant re-distribution of cells in cell cycle phases

Further, we examined the influence of P-I metalloproteinases and LAAO on cell cycle. HUVECs were treated with P-I metalloproteinases and LAAO for 12 h and analysed by flow cytometry for PI staining. The endothelial cells treated with both the proteins rearranged the distribution in G0/G1, S and G2/M phases. In comparison with control (42%), treatment of BmMP-1 and BaMP-1 showed 53% and 52% cells in G0/G1 phase respectively. Similarly, we observed the significant increase in G0/G1 phase of 54% and 63% after treating with BmLAAO and BaLAAO respectively. We also observed a decrease in the accumulation of cells in the G2/M phase when compared to control indicating reduction in the mitotic cells. These results suggested that P-I metalloproteinases and LAAO arrested the cells G0/G1 (Fig. 3B) (Additional file 3A).

### P-I metalloproteinases and LAAO from *Bothrops* spp. inhibit angiogenesis

**Figure 4.**
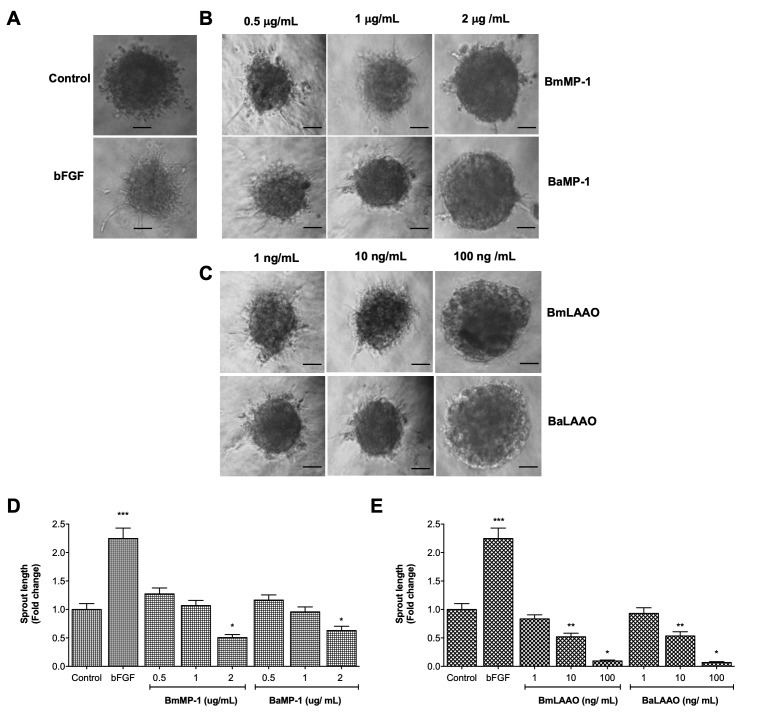
P-I metalloproteinases and LAAO inhibits endothelial sprouting. **(A, B, C)** Endothelial cells were cultured in collagen in the presence of either bFGF (10 ng/mL), or different concentrations of BmMP-1 or BaMP-1 (0.5 μg/mL, 1 μg/mL and 2 μg/mL) or BmLAAO or BaLAAO (1 ng/mL, 10 ng/mL and 100 ng/mL)/ for 24 hours and then were assessed for sprout outgrowth. Sprout length for each protein was compared to untreated control. Micrographs were captured in 10× magnification in an inverted microscope. **(D)** and **(E)** Graphical representation of modulation of angiogenesis is represented as fold change in sprout length and statistical significance is indicated as ***p < 0.001, **p < 0.01 and *p < 0.05 *versus* control. Scale = 50 μΜ.

**Figure 5.**
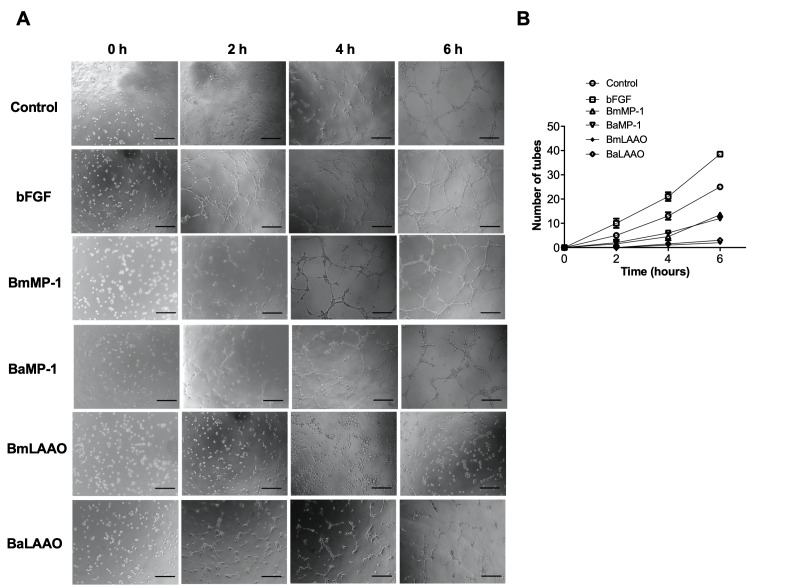
P-I metalloproteinases and LAAO inhibit endothelial tube formation. HUVECs on **(A)** Matrigel cushion were treated with BmMP-1 or BaMP-1 (2 μg/mL)/BmLAAO or BaLAAO (100 ng/mL)/bFGF (10 ng/mL) and kinetics of tube formation was counted for every 2 hours. Micrographs were captured in 10× magnification in an inverted microscope. **(B)** Graphical representation of modulation of angiogenesis is represented as fold change in tube formation. Scale = 50 μΜ.

We further tested the ability of P-I metalloproteinases and LAAO to modulate angiogenesis using *in vitro* (3D methylcellulose/collagen) and *in vivo* (dorsal skin implantation) models. Endothelial cells were cultured in 3D collagen models in the presence or absence of different concentration of BmMP-1 and BaMP-1/BmLAAO and BaLAAO and assessed for their functional role in modulating angiogenesis. As a positive control bFGF was used, which showed robust induction in the sprout formation when compared to the control (Fig. 4A). We observed inhibition of sprouts from spheroids only when treated with P-I metalloproteinases at 2 µg/mL (Fig. 4B and 4D). However, the spheroids treated with a concentration of 0.5 µg/mL and 1 µg/mL did not show any effect on inhibiting sprout formation. Further, LAAO at the concentrations of 10 ng/mL and 100 ng/mL inhibited sprout formation significantly. (Fig. 4C and 4E). To further validate the findings, we also performed matrigel assays. We observed re-arrangement of HUVECs on matrigel within 6 h upon treating with bFGF. However, P-I metalloproteinases (0.2 folds) and LAAO (0.5 folds) strongly inhibited the formation of tubes by HUVECs on Matrigel at the concentration of 2 µg/mL and 100 ng/mL respectively. While P-I metalloproteinases from both species inhibited/delayed formation of intact tubes, LAAO treatment was found to be more toxic to cells and changed the cellular phenotype (Fig. 5A and 5B).

**Figure 6.**
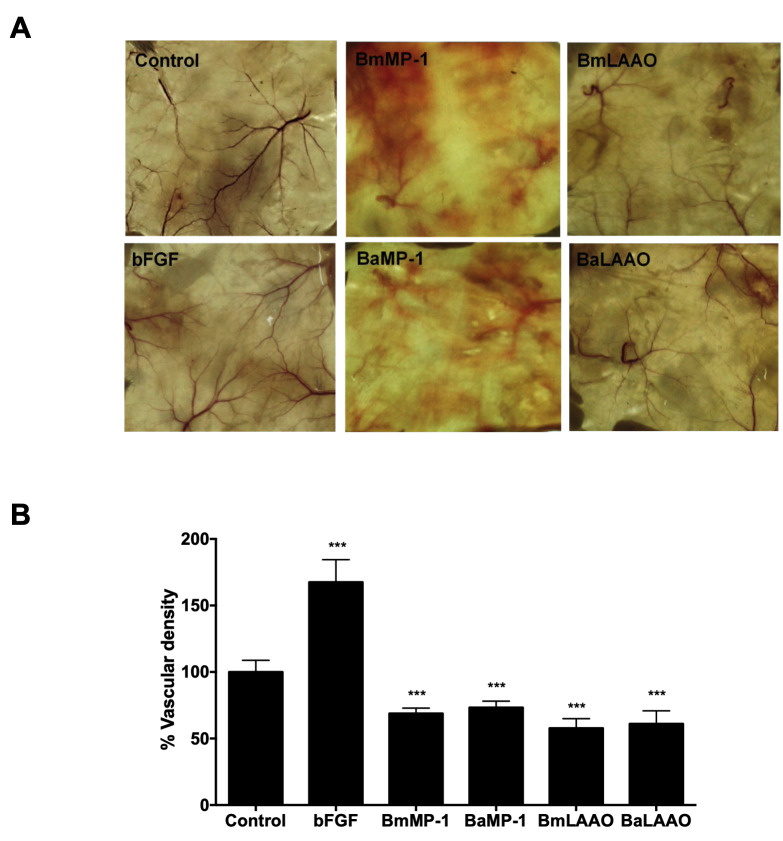
P-I metalloproteinases and LAAO inhibit angiogenesis *in vivo*. **(A)** BmMP-1 or BaMP-1 (5 μg/mL) or BmLAAO or BaLAAO (1 μg/mL) or bFGF (100 ng/mL) or PBS embedded in agarose plugs were implanted into subcutaneous space of mice and observed for five days. Degradation of capillaries was observed on skin treated with both the proteins. **(B)** The percentage of vascular density was measured and compared to control. ***p < 0.001 *versus* control.

We further investigated the effects of these proteins *in vivo* models using agarose plug assays in the mouse. Agarose was mixed with protein was placed under the subcutaneous space of mice and incubated for 5 days. Our *in vivo* data mirrored the results of *in vitro* analysis. We observed the densities of capillaries were reduced on the skin when treated with BmMP-1 and BaMP-1 (5 µg/mL) and BmLAAO and BaLAAO (1 µg/mL). These results indicated that P-I metalloproteinase and LAAO possess anti-angiogenic activities (Fig. 6A and 6B).

## Discussion

Mounting evidences have demonstrated functional characterization and therapeutic utility of metalloproteinases and LAAO from various snake venom. P-I metalloproteinases are the simple and smallest metalloproteinase protein, which exhibits the molecular mass of 20-30 kDa and contains only metalloproteinase domain [7]. These proteins harbour conserved zinc binding sequence along with Methionine-turn motifs [20]. Earlier studies have demonstrated isolation and characterization of P-I metalloproteinases from *Bothrops* spp. Patiño et al. [43] isolated P-I metalloproteinases with a molecular mass of 23.3 kDa from *B. atrox* by ion-exchange and affinity chromatography [43]. Similarly, P-I metalloproteinases isolated from *B. moojeni* by a combination of size exclusion, ion-exchange and affinity chromatography displayed molecular mass of 22.6 kDa protein [19]. In our present study, the P-I metalloproteinases isolated upon combining size exclusion and ion-exchange chromatography from *B. moojeni* and *B. atrox* showed molecular mass of ~25 kDa as observed in SDS PAGE.

P-I metalloproteinases were extensively characterized by their catalytic activity on casein and fibrinogen. P-I metalloproteinases (BaP1) isolated from *B. asper* degraded the casein at 17.8 units/mg protein [44]. P-I metalloproteinases (Bmoo FIBMP-I) from the venom of *B. moojeni* exhibited pH dependent catalytic activity on azocasein with K_m_ = 14.59 mg/mL ± 4.610 and V_max_ = 0.4596 Uh^−1^ nmol^−1^ ± 0.1031 [45]. Kayano et al. [40] isolated P-I metalloproteinases from *B. brazili* that showed activity at different concentrations and were inhibited by EDTA and DTT, but not by PMSF. Metalloproteinases isolated from *Lachesis muta rhombeata* snake venom showed maximum activity on azocasein at pH 7.0 - 9.0 and were also inhibited by EDTA but no effect with PMSF [46]. P-I metalloproteinases isolated from *B. moojeni* [47, 48], *B. asper* [49] and *B. atrox* [43] cleaved Aα chain of fibrinogen. Further, proteomic analysis of *Bothrops* snake venom demonstrated an intense gelatinolytic activity around 25 kDa region [48, 50]. In agreement with previous studies and as summarized in Table 1, in our study P-I metalloproteinases purified from *Bothrops* spp. also showed significant ability to digest casein and possessed gelatinolytic and fibrinogenolytic activity.

The class of LAAO molecules was first identified by Zeller and Maritiz (1945) from the venom of *Vipera aspis* [51]. Singer and Kearney [52] first isolated LAAO from *Agkistrodon piscivorus* venom and the crystal structure of LAAO from *Crotalus adamanteus* venom was obtained by Wellner and Meister in 1960s [53]. The molecular weight of LAAO as reported in earlier studies ranged from 38 kDa to 67 kDa. Earlier studies have purified and performed biochemical characterization of LAAO proteins. LAAO isolated from *B. atrox* by Costal-Oliveira et al. [32] by three chromatographic process molecular size exclusion, ion-exchange and affinity chromatography showed molecular mass of 57 kDa protein. Further, Stábeli et al. [54] isolated LAAO (64.8 kDa) from *B. moojeni* by ion-exchange chromatography and phenyl-sepharose chromatography. The LAAO isolated from both *B. moojeni* and *B. atrox* in the present study exhibited molecular mass of ~58 kDa protein as observed on SDS PAGE which is in the agreement with the previous studies [32,54,55].

LAAO isolated from various snake venom for over the years have demonstrated differential activity towards L-amino acids. LAAO isolated from *B. atrox* showing high catalytic activity against M>L>F>W>Y>I at a concentration of 15 µg/mL [56]. Further, LAAO (Balt-LAAO-1) from *B. alternatus* showed catalytic activity only towards P>T>M>L [57], but LAAO (Bpir-LAAO-1) isolated from *B. pirajai* at a concentration of 2U exhibited catalytic activity on hydrophobic and long chain amino acids F>Y>W>L>M>I>V>H [58]. Although, the protein homology of the LAAO from *Bothrops* spp. ranged from 80% to 90%, activity of the proteins on amino acids varied as observed in earlier studies. This suggested that the structural makeup of LAAO protein might impact activity and substrate specificity.

The evolution of snake venom molecules over the millions of years facilitated in capturing the prey and defence from predators. Majority of the snake venoms have been well characterized and comprises mixture of distinct array of proteins and peptides results in varied toxicological and pharmacologically effects [59]. The main pharmacological effects of snake venom are broadly classified as hemotoxic, cytotoxic and neurotoxic [60] caused by major proteins, either alone or combination of LAAO, phospholipase A_2,_ snake venom serine proteinases, SVMPs and 3 finger toxins occurs during envenomation. The metalloproteinases in venom causes local and systematic hemorrhage, affecting the blood vessels [18]. These hemorrhagic enzymes may directly breakdown the basement components and their key peptide bonds, affecting the endothelial cell interaction between the basement membrane predominantly type IV collagen and perlecan [61]. *In vivo,* this may facilitate morphological and functional alteration of endothelial cells affecting extravasation thereby forming gaps between the endothelial cells leading to divergent fluid shear stress [62]. LAAO in the venom majorly causes hemorrahge and edema promoting the degradation of matrix proteins of endothelial cells and increasing the vascular permeability which may fluid leakage from the capillary vessels to interstitial space of tissues [57]. LAAO causes cytotoxicity on endothelial cells by necrosis and apoptosis. Necrosis is caused by direct action of the protein or catabolic products of plasma membrane which promotes the degeneration of the cells and apoptosis is caused by ROS production [4]. Taken together, these findings suggest that during envenomation, snake venom components induce cytotoxic effects on various cell types including endothelial cells, neuronal cells, myoblasts and epithelial cells and might contribute to delayed tissue regeneration and associated wound healing.

Snake venom proteins and peptides have been extensively explored for their properties to target pathological angiogenesis. The previous studies have demonstrated serine proteinases to facilitate significant neovascularization *via* PI3K/Akt pathway [39]. Similarly, proteins inhibiting angiogenesis are of pharmaceutical interest particularly to inhibit to neovascularization in pathological conditions associated with excessive angiogenesis such as in tumours, diabetic retinopathy and arthritis. Hence, in the present study, we have demonstrated that P-I metalloproteinases from *B. atrox* and *B. moojeni* and LAAO from *B. moojeni* and *B. atrox* showed anti-angiogenic properties.

SVMPs have been demonstrated as versatile toxins, which target hemostatic system such as clotting proteins, endothelial cells, basement membrane proteins and platelets [63]. Earlier studies have demonstrated that SVMP activated apoptotic pathways. A metalloproteinase (jararhagin) isolated from *B. jararaca* induced apoptosis of endothelial cells *via* anoikis [24,64]. The catalytic activity of the protein is reported to affect the cytoskeleton dynamics such as cell retraction, followed by alteration of actin network, reduced association of focal adhesion kinase (FAK) to actin and also indicated to interfere with the focal adhesion contacts of tyrosine phosphorylated proteins. It was observed that the morphological changes on endothelial cells resulted in pro-caspase-3 activation leading to apoptosis and alteration in the ratio between Bax/Bcl-xL [64]. BaP1 (metalloproteinase-1) from *B. asper* venom [51,65] induced apoptosis in endothelial cells *via* activated pro-caspase 8 and c-FLIPL up-regulation [66] . However, jararhagin [64] and halysase [67] induced apoptosis through Bax and Bcl-xL expression, leading to activation of the extrinsic pathway [66]. Further, graminelysin (metalloproteinase-1 isolated from *Trimeresurus gramineus* venom) [68] triggered apoptosis by its catalytic activity on endothelial cells which occurred before cell detachment, subsequently activating the caspase-3 and the reduction in the ratio of Bcl-2/Bax [69]. The proteins associated with adherens junction were cleaved with a significant reduction in the levels of α-catenin, which disrupted the association with the actin cytoskeleton and interfere with the endothelial cell extracellular signals [69]. BpMP-II (P-I metalloproteinases) isolated from *B. pauloensis* influenced adhesive and tube forming properties of rat thymic endothelial cells suggesting, anti-angiogenic property of SVMPs [70]. Corroborating previous studies, P-I metalloproteinases from both *B. atrox* and *B. moojeni* induced apoptosis in endothelial cells. However, the concentrations of these proteins used in our study was significantly lower (2 μg/mL) against earlier studies which ranged from 20-200 μg/mL [60-70]. Interestingly, we also observed that P-I-metalloproteinase-induced apoptosis was more intense in HUVEC than in fibroblasts and HepG2.

LAAO in the venom, majorly responsible for apoptosis in endothelial cells by producing H_2_O_2_ and inducing caspase-mediated apoptosis driven by ROS, activating caspase-3 and caspase-9 [71,72]. Apoxin I (LAAO from *Crotalus atrox*), showed apoptotic effect on HUVECs, A2780, HL-60 and NK-3 (rat endothelial cells) by inducing the condensation and fragmentation of DNA [72]. Wei et al. [73] isolated LAAO (BF-LAAO) from the venom of *Bungarus fasciatus*, inhibited the growth and induced the apoptosis in HUVECs by dose depended manner with an IC50 value of 2.8 mg/L. LAAO-II isolated from *B. jararacassu* significantly reduced the viability and survival and further, induced DNA damage in human endothelial cells upon increasing ROS levels [30]. Concentrations of LAAO used in our study were also lower than that of earlier studies [30,71-73] and similarly to P-I metalloproteinases, LAAO induced cytotoxic effects were more prominent in endothelial cells than fibroblasts.

## Conclusion

In the present study, we have isolated P-I metalloproteinases and LAAO from *B. moojeni* and *B. atrox*, and extensively characterized them based on their enzymatic activities. P-I metalloproteinases and LAAO arrested the cells in G0/G1 phase of cell cycle and also led to cytotoxic activity in endothelial cells by inducing necrosis/late apoptotic mode of cell death. We further demonstrated that both proteins were able to distress the endothelial cells *in vitro* and significantly inhibited the growth of the capillaries *in vivo*. Our findings suggest that metalloproteinases and LAAO from venoms cause cytotoxicity to endothelial cells and might inhibit tissue regeneration and subsequently delay wound healing. Such anti-vascular effects of these proteins can be further explored to design therapeutic strategies to inhibit angiogenesis. However, further studies are essential to investigate the underlying molecular mechanisms and explore the therapeutic implications of these proteins.

## Supplementary material

The following online material is available for this article:

Additional file 1. Inhibitory activity of LAAO. The effect of inhibitors on **(A)** BmLAAO or **(B)** BaLAAO activity was measured in the presence or absence of divalent metals CaCl_2_, MgCl_2_, ZnCl_2_ and HgCl_2_ (all 10 mM) and amino acid derivative N-acetyl cysteine (NAC) (5 mM). Data are represented as percentage of untreated control and statistical significance is indicated by asterisk ***p < 0.001.

Additional file 2. Influence of P-I metalloproteinases and LAAO from *Bothrops* venom on cell survival. **(A)** and **(B)** Endothelial cells, **(C)** HepgG2 and fibroblast cells were treated with either BmMP-1 or BaMP-1 or BmLAAO or BaLAAO and mitomycin for 24 hours in normal culture conditions and cells were processed for MTT assay. Data are represented as percentage of untreated control cells *versus* treated in each cell type. Statistical significance was denoted by asterisk. ***p < 0.001, **p < 0.01, *p < 0.05 *versus* control.

Additional file 3. Influence of P-I metalloproteinases and LAAO on apoptosis and cell cycle. The endothelial cells were treated with BmMP-1 or BaMP-1 (2 µg/mL) and BmLAAO or BaLAAO (100 ng/mL) and processed for flow cytometry analysis. **(A)** Cells were stained with annexin V conjugated with FITC and propidium iodide and analysed in a flow cytometer. **(B)** After treating cells as indicated above, they were fixed in ethanol and stained with PI and analysed by flow cytometry.
