# Supplementary material for: P-I metalloproteinases and L-amino acid oxidases from Bothrops species inhibit angiogenesis
Source: J Venom Anim Toxins Incl Trop Dis. 2021 Aug 18;27:e20200180. doi: 10.1590/1678-9199-JVATITD-2020-0180 (PMC8381740; doi:10.1590/1678-9199-JVATITD-2020-0180)
Supplement: Additional file 1. [file 1678-9199-jvatitd-27-e20200180-s1.pdf]

J Venom Anim Toxins incl Trop Dis
https://doi.org/10.1590/1678-9199-JVATITD-2020-0180

Supplementary Material to "P-I metalloproteinases and L-amino acid oxidases from *Bothrops* species inhibit angiogenesis"

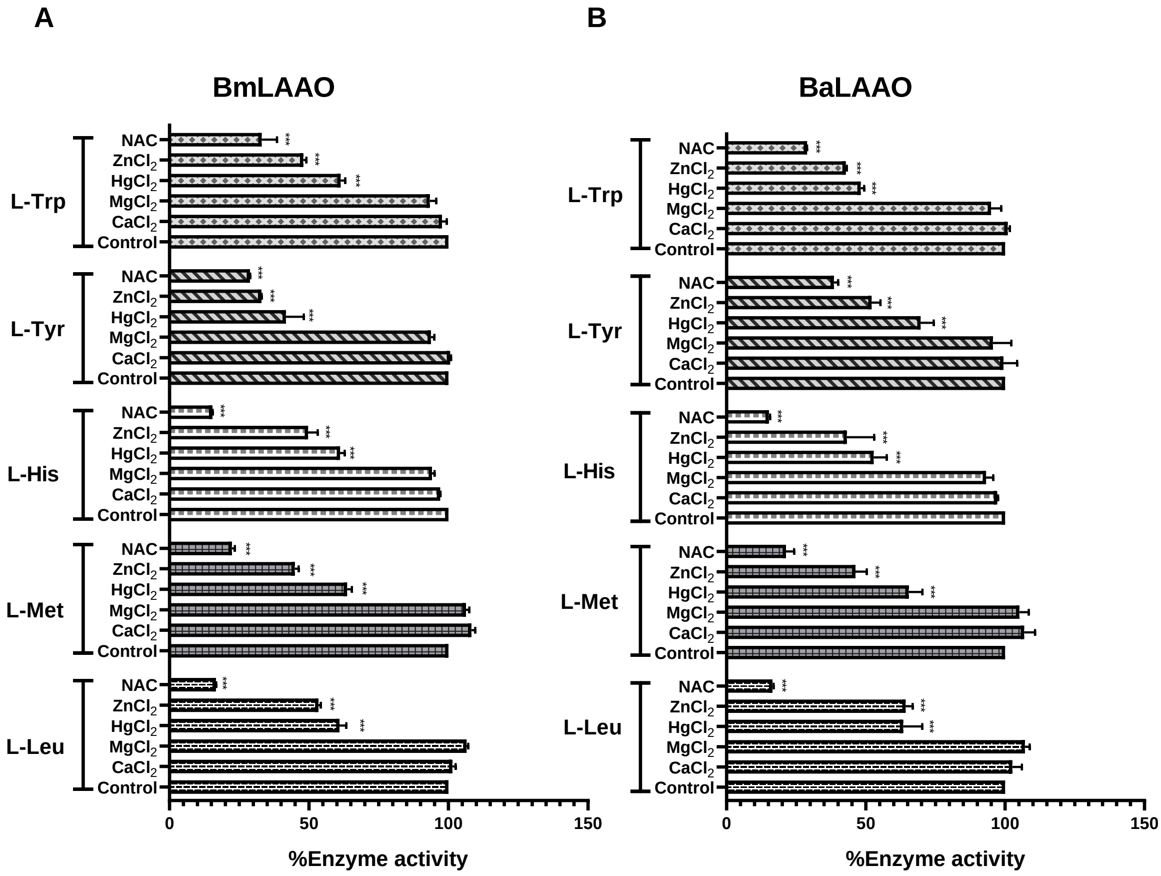

Additional file 1. Inhibitory activity of LAAO. The effect of inhibitors on (**A**) BmLAAO or (**B**) BaLAAO activity was measured in the presence or absence of divalent metals $CaCl_2$, $MgCl_2$, $ZnCl_2$ and $HgCl_2$ (all 10 mM) and amino acid derivative N-acetyl cysteine (NAC) (5 mM). Data are represented as percentage of untreated control and statistical significance is indicated by asterisk ***$p < 0.001$.
