# Supplementary material for: P-I metalloproteinases and L-amino acid oxidases from Bothrops species inhibit angiogenesis
Source: J Venom Anim Toxins Incl Trop Dis. 2021 Aug 18;27:e20200180. doi: 10.1590/1678-9199-JVATITD-2020-0180 (PMC8381740; doi:10.1590/1678-9199-JVATITD-2020-0180)
Supplement: Additional file 2. [file 1678-9199-jvatitd-27-e20200180-s2.pdf]

J Venom Anim Toxins incl Trop Dis
https://doi.org/10.1590/1678-9199-JVATITD-2020-0180

Supplementary Material to "P-I metalloproteinases and L-amino acid oxidases from *Bothrops* species inhibit angiogenesis"

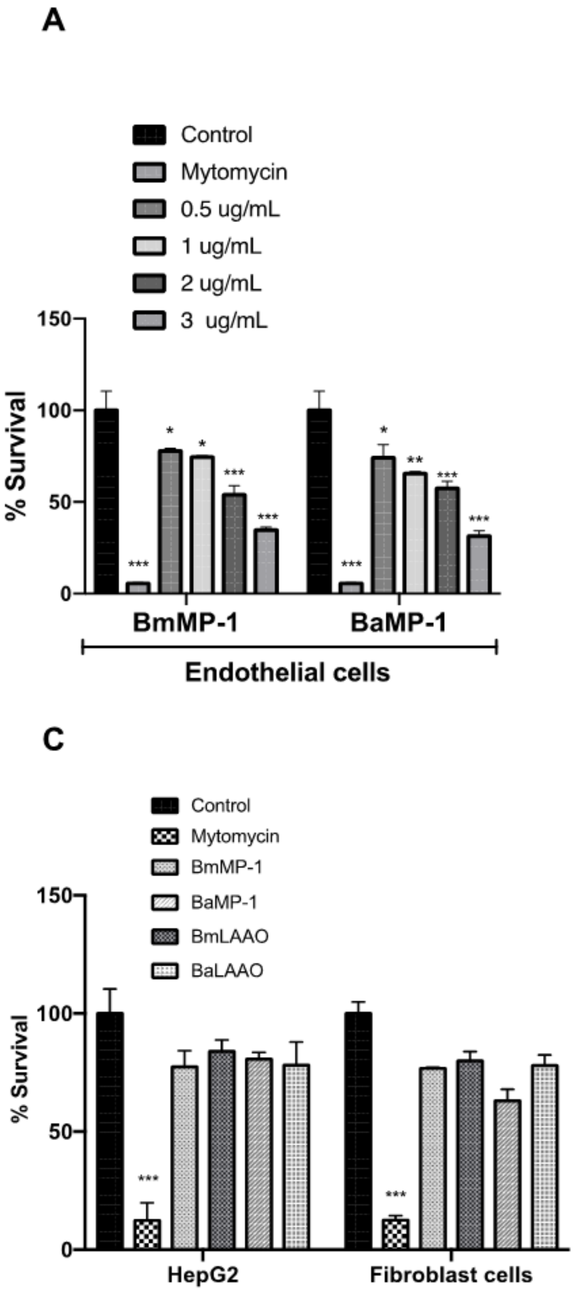

Additional file 2. Influence of P-I metalloproteinases and LAAO from *Bothrops* venom on cell survival. (**A**) and (**B**) Endothelial cells, (**C**) HepgG2 and fibroblast cells were treated with either BmMP-1 or BaMP-1 or BmLAAO or BaLAAO and mitomycin for 24 hours in normal culture conditions and cells were processed for MTT assay. Data are represented as percentage of untreated control cells *versus* treated in each cell type. Statistical significance was denoted by asterisk. ***p < 0.001, **p < 0.01, *p < 0.05 *versus* control.
