# Supplementary material for: P-I metalloproteinases and L-amino acid oxidases from Bothrops species inhibit angiogenesis
Source: J Venom Anim Toxins Incl Trop Dis. 2021 Aug 18;27:e20200180. doi: 10.1590/1678-9199-JVATITD-2020-0180 (PMC8381740; doi:10.1590/1678-9199-JVATITD-2020-0180)
Supplement: Additional file 3. [file 1678-9199-jvatitd-27-e20200180-s3.pdf]

J Venom Anim Toxins incl Trop Dis
https://doi.org/10.1590/1678-9199-JVATITD-2020-0180

Supplementary Material to "P-I metalloproteinases and L-amino acid oxidases from *Bothrops* species inhibit angiogenesis"

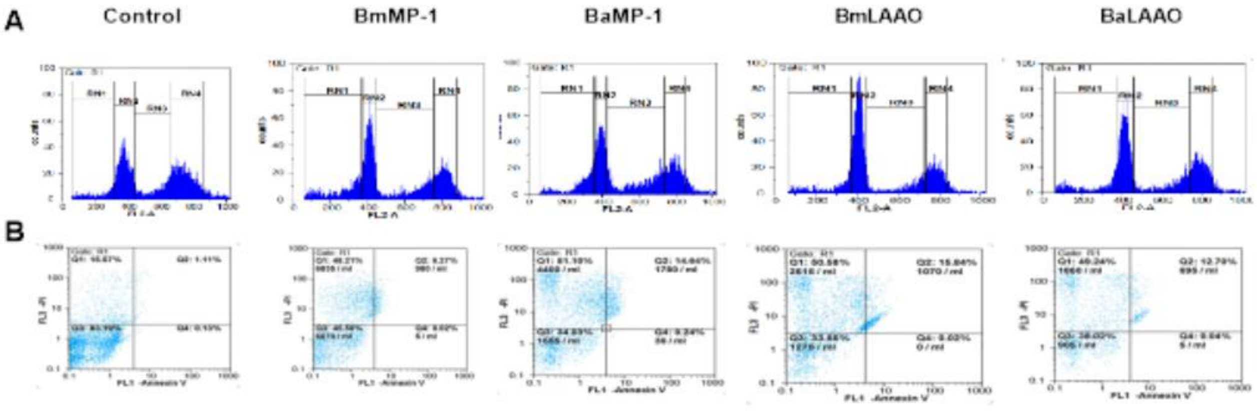

Additional file 3. Influence of P-I metalloproteinases and LAAO on apoptosis and cell cycle. The endothelial cells were treated with BmMP-1 or BaMP-1 (2 µg/mL) and BmLAAO or BaLAAO (100 ng/mL) and processed for flow cytometry analysis. (**A**) Cells were stained with annexin V conjugated with FITC and propidium iodide and analysed in a flow cytometer. (**B**) After treating cells as indicated above, they were fixed in ethanol and stained with PI and analysed by flow cytometry.
